# SUMOylation of Alpha-Synuclein Influences on Alpha-Synuclein Aggregation Induced by Methamphetamine

**DOI:** 10.3389/fncel.2018.00262

**Published:** 2018-08-24

**Authors:** Lin-nan Zhu, Hong-hua Qiao, Ling Chen, Le-ping Sun, Jia-liang Hui, Yong-ling Lian, Wei-bing Xie, Jiu-yang Ding, Yun-le Meng, Bo-feng Zhu, Ping-ming Qiu

**Affiliations:** ^1^School of Forensic Medicine, Southern Medical University, Guangzhou, China; ^2^First Clinical Medical College, Southern Medical University, Guangzhou, China; ^3^Department of Anatomy, Zunyi Medical College, Zunyi, China

**Keywords:** methamphetamine, SUMOylation, alpha-synuclein, aggregation, degradation

## Abstract

Methamphetamine (METH) is an illegal and widely abused psychoactive stimulant. METH abusers are at high risk of neurodegenerative disorders, including Parkinson’s disease (PD). Previous studies have demonstrated that METH causes alpha-synuclein (α-syn) aggregation in the both laboratory animal and human. In this study, exposure to high METH doses increased the expression of α-syn and the small ubiquitin-related modifier 1 (SUMO-1). Therefore, we hypothesized that SUMOylation of α-syn is involved in high-dose METH-induced α-syn aggregation. We measured the levels of α-syn SUMOylation and these enzymes involved in the SUMOylation cycle in SH-SY5Y human neuroblastoma cells (SH-SY5Y cells), in cultures of C57 BL/6 primary mouse neurons and in brain tissues of mice exposure to METH. We also demonstrated the effect of α-syn SUMOylation on α-syn aggregation after METH exposure by overexpressing the key enzyme of the SUMOylation cycle or silencing SUMO-1 expression *in vitro*. Then, we make introduced mutations in the major SUMOylation acceptor sites of α-syn by transfecting a lentivirus containing the sequence of WT α-syn or K96/102R α-syn into SH-SY5Y cells and injecting an adenovirus containing the sequence of WT α-syn or K96/102R α-syn into the mouse striatum. Levels of the ubiquitin-proteasome system (UPS)-related makers ubiquitin (Ub) and UbE1, as well as the autophagy-lysosome pathway (ALP)-related markers LC3, P62 and lysosomal associated membrane protein 2A (LAMP2A), were also measured in SH-SY5Y cells transfected with lentivirus and mice injected with adenovirus. The results showed that METH exposure decreases the SUMOylation level of α-syn, although the expression of α-syn and SUMO-1 are increased. One possible cause is the reduction of UBC9 level. The increase in α-syn SUMOylation by UBC9 overexpression relieves METH-induced α-syn overexpression and aggregation, whereas the decrease in α-syn SUMOylation by SUMO-1 silencing exacerbates the same pathology. Furthermore, mutations in the major SUMOylation acceptor sites of α-syn also aggravate α-syn overexpression and aggregation by impairing degradation through the UPS and the ALP *in vitro* and *in vivo*. These results suggest that SUMOylation of α-syn plays a fundamental part in α-syn overexpression and aggregation induced by METH and could be a suitable target for the treatment of neurodegenerative diseases.

## Introduction

Methamphetamine (METH), a highly addictive amphetamine-typed stimulant, is commonly abused worldwide (Krasnova and Cadet, 2009; Carvalho et al., 2012). Multiple studies, including ours, have shown that dopamine depletion, oxidative stress, mitochondrial functional impairment, endoplasmic reticulum stress and axonal transport barriers may be involved in the main mechanism of METH neurotoxicity (Cadet and Krasnova, 2009; Yamamoto et al., 2010; Zhang et al., 2013; Chen C. et al., 2016). The principal target of METH is the central nervous system, in particular dopaminergic neurons (Mark et al., 2004; Li et al., 2017). Furthermore, METH causes pathological changes similar to neurodegenerative diseases like Parkinson’s disease (PD; Garwood et al., 2006; Morrow et al., 2011). In both laboratory animals and humans, METH exposure increases the expression of alpha-synuclein (α-syn) in the striatum and prefrontal cortex (Jiang et al., 2014; Wang and Witt, 2014; Flack et al., 2017). α-Syn, a natively unfolded neuronal protein enriched in presynaptic terminals, has been involved in several neurodegenerative diseases, including PD and dementia with Lewy bodies (LBs). Furthermore, α-syn accumulates and aggregates during the pathogenic process (Lee and Lee, 2002; Kahle, 2008; Vekrellis et al., 2011). It was reported that α-syn aggregates accumulate throughout axons and impair retrograde axonal transport (Volpicelli-Daley et al., 2014). Other study said that a majority of α-syn aggregated in LBs cases were located at the presynapses. They visualized tiny α-syn was rich in the cortex of neurodegenerative diseases patients (Schulz-Schaeffer, 2010). Posttranslational modifications including phosphorylation, nitrosylation and ubiquitination of α-syn have been found to play roles in α-syn accumulation and aggregation (Fernández et al., 2014; Alexopoulou et al., 2016; Kumar P. et al., 2017; Kumar R. et al., 2017; Zhong et al., 2017).

The small ubiquitin-related modifier (SUMO) is covalently conjugated to lysine residues in a wide range of substrate proteins, regulating the functional properties of the modified protein (Cajee et al., 2012). Similar to ubiquitination, the SUMOylation cycle involves a series of enzymes in a multistep process. E1-activating enzymes, such as SAE1 and SAE2, initiate the SUMOylation cycle. Next, UBC9 as the only known specific E2-conjugating enzyme receives the activated SUMO and conjugates it to a substrate protein, occasionally with help of an E3 ligase, including the PIAS family members, the polycomb group protein Pc2 and the RanBP2 (Vijayakumaran et al., 2015). The level of SUMO conjugation coincides with the expression level of UBC9 (Lee et al., 2007). SUMOylation is also easily reversible through the activity of SUMO-specific proteases (SENPs; Cimarosti et al., 2012). Several aggregation-prone proteins implicated in neurodegeneration like α-syn are reported to be SUMOylated (Dorval and Fraser, 2006), and SUMOylation-deficient mutants proteins revealed an enhanced tendency to aggregate (Krumova et al., 2011). Nevertheless, the effects of SUMOylation on α-syn are complicated, with reports supporting negative as well as positive impacts. Currently, the exact contribution of SUMOylation to the effects of METH on α-syn remains unclear. Of the four mammalian SUMO isoforms identified, we would focus on the SUMO-1 isoform in our study, because SUMO-1 has been found to be relevant to pathological inclusion bodies in neurodegenerative diseases.

Significant protein deposition can occur in the intracellular space in either or both of two ways: the propensity of proteins to aggregate can be modulated, or the clearance of protein aggregates by the cellular degradation machinery can be affected. Protein deposition has been linked to defects in the two primary protein catabolic pathway, the ubiquitin-proteasome system (UPS) and the autophagy-lysosome pathway (ALP; Mitra et al., 2009; Ebrahimi-Fakhari et al., 2011, 2012; Wang et al., 2013). The universal consensus is that, although the UPS only degrades small, soluble α-syn oligomers, the ALP is essential in pathological conditions. Ubiquitin (Ub), a small regulatory protein, is conjugated to substrate proteins, applying these proteins to be degraded through the UPS. UbE1 (Uba1) is required for ubiquitination because it initiates the first key step of the UPS by catalyzing Ub activation; therefore, UbE1 inactivation completely blocks ubiquitination (Pelzer et al., 2007). Although an amount of proteins is degraded by the UPS, some cytosolic proteins are degraded by a more distinctive pathway: the ALP. The ALP is a lysosome-mediated catabolic mechanism in charge of degradation of damaged or dysfunctional proteins and recycling of their effective constituent (Zheng and Bao, 2017; Kan et al., 2018; Lassen and Xavier, 2018). Several protein complexes regulate the ALP. For example, lipidation and redistribution of the cytoplasmic protein LC3 towards the phagophore conduces to its elongation around the cargo to be engulfed; and an autophagic receptor such as p62/SQSTM1 recognizes the cargo. When the autophagic flux is damaged, the cargo is not degraded, and resulting in p62/SQSTM1 accumulation. Lysosomal associated membrane protein 2A (LAMP2A), interacts with other proteins and, forms a multiprotein complex to regulate the translocation of the substrate into the lumen of the lysosome, where it could be degraded. A deficiency in the UPS or the ALP in neuronal cells can accelerate the development of neurodegenerative diseases (Bourdenx et al., 2017).

The objective of this study was to determine whether METH impacts the α-syn SUMOylation and how the SUMOylation of α-syn influences METH-induced α-syn aggregation in neuronal cells. We hypothesized that METH affects SUMOylation of α-syn and that, in turn, α-syn SUMOylation may reciprocally mediate METH-induced α-syn aggregation. Therefore, we measured the levels of α-syn SUMOylation and related enzymes involved *in vitro* and *in vivo* following METH exposure to verify the hypothesis. This study also investigated the effect of α-syn SUMOylation on the α-syn aggregation after METH exposure by overexpressing the key enzyme in the SUMOylation cycle or silencing SUMO-1 expression *in vitro*. Then, we made introduced mutations in the main SUMOylation acceptor sites of α-syn, and measured the UPS- and ALP-related markers. The results showed that METH exposure decreased the SUMOylation level of α-syn and reduced the UBC9 level. The change in α-syn SUMOylation regulated METH-induced α-syn overexpressing and aggregation. Furthermore, mutations in the major SUMOylation acceptor sites of α-syn aggravate α-syn overexpressing and aggregation by impairing degradation through the UPS and the ALP *in vitro* and *in vivo*. The study also provides a potential target for gene therapy for neurodegenerative diseases, even those caused by METH abuse.

## Materials and Methods

### Animal Protocol

Healthy adult male C57 BL/6 mice were purchased from Laboratory Animal Center of Southern Medical University (Guangzhou, China) and housed singly in tub cages in a temperature-controlled room on a 12-h light-12-h dark cycle.

The mice were divided randomly into the saline control group and the subacute exposure group (*n* = 10/group). Mice in the control and subacute exposure groups were exposed to saline or 15 mg/kg METH (>99% purity; National Institutes for Food and Drug Control, Guangzhou, China), respectively, via eight intraperitoneal (i.p.) injections at 12-h intervals. Based on several previous studies, this subacute exposure paradigm was relevant to short-term exposure in humans because the measured concentrations of METH in the blood and brain of mice at 1 h after the last injection were in the range of reported blood concentrations (0.6–5 mg/ml [4–30 mM]) in humans (Winek et al., 2001; Du et al., 2017). Mice were euthanized at 24 h after the last injection. Brain samples (prefrontal cortex and striatum tissues) were removed quickly, dissected on ice and stored at −80°C. We selected the prefrontal cortex and striatum because α-syn level was significantly increased in those two brain regions (Jiang et al., 2014; Wang and Witt, 2014; Flack et al., 2017). All animal procedures were performed according to the National Institutes of Health (NIH) Guide for the Care and Use of Laboratory Animals and were pre-approved in advance by the Institutional Animal Care and Use Committee at the Southern Medical University.

### Cell Culture

SH-SY5Y human neuroblastoma cells, SH-SY5Y cells were purchased from the Shanghai Cell Bank of the Chinese Academy of Sciences (Shanghai, China). This cell line was selected as an *in vitro* model because it was commonly used to study neurotoxicology of many toxicants, including METH (Huang et al., 2015; Speen et al., 2015). SH-SY5Y cells were cultured in DMEM/F12 (1:1) containing fetal bovine serum (Gibco, Grand Island, NY, USA). SH-SY5Y cells were initially exposed to METH concentrations of 0.5 mM, 1.0 mM, 1.5 mM, 2.0 mM and 2.5 mM for 24 h, and the concentration of 2.0 mM for SH-SY5Y cells was selected for subsequent experiments on the basis of the LC25 of METH in this cell type (Huang et al., 2015). Then, we exposed SH-SY5Y cells to 2.0 mM METH for 0 h, 2 h, 4 h, 8 h, 12 h and 24 h. These *in vitro* concentrations are consistent with earlier studies from our lab (Li et al., 2017) and other groups (Ferrucci et al., 2017; Uno et al., 2017), thus allowing comparisons between studies.

### Primary Cultures of Prefrontal Cortical and Striatal Neurons From C57 BL/6 Mice

Primary prefrontal cortical and striatal neuronal cultures were prepared from C57 mice as previously described (Danzer et al., 2012; Dong et al., 2012; Lepsch et al., 2015). Briefly, E18 C57 mouse embryos were harvested by cesarean section from an anesthetized pregnant C57 mouse after the skin cleaned with 75% ethanol, and then the embryos were cleaned in a Petri dish containing CMF-HBSS (calcium- and magnesium-free Hank’s balanced salt solution, 4°C). Fetal brains were isolated under a dissection microscope and transferred to another Petri dish containing CMF-HBSS (4°C); then, the prefrontal cortex and striatum were dissected out and transferred to a new dish containing DMEM/F12 (1:1; 4°C). The dissected tissues were minced into 1-mm^3^ pieces, and then the excess medium was removed. The digestion step was performed by adding 3–5 ml of 0.25% trypsin-EDTA (Gibco), and the cells was incubated at 37°C in a humidified atmosphere for 10 min. Subsequently, 10 ml of DMEM/F12 (1:1) supplemented with FBS was added to Petri dish to terminate the digestion. The mixture containing individual cells was collected and then centrifuged at 1,000 rpm for 5 min. The supernatant was removed, and the tissue pellet was resuspended in neurobasal medium containing 2% B-27, 1% Glutamax-100X and 5 mM glutamate (Gibco). Cells were plated in six-well plates or confocal dishes, which were precoated with 0.01% poly-L-lysine (Sigma, St. Louis, MO, USA). The medium was replaced 3 days after plating and every 2 days thereafter with Neurobasal medium supplemented with 2% B-27 and 1% Glutamax-100X. Neuronal culture neurons were maintained for up to 5–7 days and then used for subsequent experiments. Primary cultures of prefrontal cortical and striatal neurons were exposed to 0.2 mM, 0.4 mM, 0.6 mM, 0.8 mM and 1.0 mM METH for 24 h. These concentrations were based on the LC25 (0.58 mM) of METH in primary cultured neurons as measured in our lab (Chen R. et al., 2016; Xu et al., 2017). Furthermore, these concentrations are semblable between used in the SH-SY5Y cells and primary cultured neurons.

### Immunofluorescence Labeling

All samples were prepared using phosphate-buffered saline (PBS) supplemented with 10% goat serum and 0.1% Triton X-100. Next, the samples were incubated with blocking buffer (5% BSA in TBST, 1 TBS + 0.1% TWEEN 20 buffer) for 45 min at room temperature and then with anti-SUMO-1 (#4930, 1:100 dilution, Cell Signaling Technology, Boston, MA, USA) and anti-α-syn (#2628, 1:100 dilution, Cell Signaling Technology) primary antibodies, at 4°C overnight. After washed with PBS, the samples were incubated with secondary antibody [goat anti-rabbit IgG/fluorescein isothiocyanate (FITC) antibody (bs-0295G-FITC, 1:100 dilution, Bioss, Beijing, China)] for 1 h at room temperature. VECTASHIELD Antifade Mounting Medium with DAPI (H-1200, VECTOR, Burlingame, CA, USA) was used for nuclear labeling. Photomicrographs were captured using a fluorescence microscopy (A1+/A1R+, Nikon, Tokyo, Japan). All digital images were processed using the same settings to improve the contrast.

### Immunohistochemistry

The prefrontal cortex and striatum were fixed in buffered formalin fixative. Frozen tissue sections (5 mm in thickness) were sliced using a freezing microtome (CM1900, Leica, Wetzlar, Germany). The sections were pretreated in 0.01 M citrate buffer, pH 6.0, by hydrated autoclaving in a humid atmosphere for 10 min and incubated with blocking buffer for 40 min at room temperature. These sections were incubated with anti-α-syn (Syn204) antibody (#2647, 1:50 dilution, Cell Signaling Technology) or anti-SUMO-1 antibody (#4930, 1:50 dilution, Cell Signaling Technology) at 4°C overnight. Thereafter, these sections were incubated with the secondary antibody for 30 min at room temperature. After washed with PBS, the samples were developed with 3,3′-diaminobenzidine (DAB) kits (ZSGB-BIO, Beijing, China) for 2–5 min at room temperature. For the measurement of protein level, images were captured with a digital camera (DXM1200F, Nikon, Tokyo, Japan) connected to a microscope (Eclipse 80i, Nikon) with a 40× objective.

### Western Blot

The samples were lysed in radioimmunoprecipitation assay (RIPA) buffer with protease inhibitors at 4°C for 30 min. Protein concentrations were measured with the BCA-100 Protein Quantitative Analysis kit (Biocolors, Shanghai, China). Protein samples (10 mg) were separated by sodium dodecyl sulfate polyacrylamide gel electrophoresis (SDS-PAGE) and transferred onto polyvinylidene difluoride (PVDF) membranes (Millipore, Billerica, MA, USA). The membranes were blocked at room temperature for 1 h in blocking buffer, followed by incubation with diluted primary antibodies with gentle shaking at 4°C overnight. The anti-SUMO-1 antibody (#4930; 1:1,000 dilution), anti-α-syn antibody (#2642; 1:1,000 dilution), anti-LC3B antibody (#3868; 1:1,000 dilution), anti-SQSTM1/p62 antibody (#5114, 1:1,000 dilution), anti-Ubiquitin antibody (#3933, 1:1,000 dilution) and anti-UBE1a/b (#4891, 1:1,000 dilution) were purchased from Cell Signaling Technology. Anti-aggregated α-syn, clone 5G4 (#MABN389; 1:1,000 dilution) which was used to measure the levels of the aggregated α-syn protein, was purchased from Millipore. The anti-LAMP2A antibody (ab18528; 1:1,000 dilution) was purchased from Abcam. Membranes were washed with TBST buffer and then incubated with the corresponding horseradish peroxidase (HRP)-conjugated secondary antibodies at room temperature for 1 h. Membranes were developed with ECL Plus chemiluminescent Western blot detection reagents, and band intensity signals were quantitated with a Gel-Pro analyzer (Media Cybernetics Inc., Rockville, MD, USA). Blot images were analyzed and peak areas integrated using the ImageJ software. β-Actin was used as a reference control. For each protein of interest, we conducted three independent experiments, and selected the representative band shown in this manuscript.

### RT-QPCR

Total RNA was extracted using RNAisoPlus Kit from METH-treated SH-SY5Y cells. Then, cDNA was reversely transcribed from 1 mg of total RNA using the PrimeScriptTM RT reagent Kit and SYBRs Premix ExTaqTM Kit. Primers were designed by Takara Bio Co., Limited (Dalian, China). All the sequences of protein primers were listed as Supplementary Table S1.

### Co-immunoprecipitation

The samples were lysed in RIPA buffer supplemented with protease inhibitors at 4°C for 30 min. The protein suspensions were incubated with an anti-SUMO-1 antibody (#4930; 1:50 dilution, Cell Signaling Technology) or control IgG (A7016, Beyotime, Shanghai, China) with gentle shaking at 4°C overnight, and then Protein A/G (P2012, Beyotime) was added and incubated with gentle shaking at 4°C for 1–3 h. Each mixture was centrifuged at 2,500 rpm for 5 min at 4°C. Subsequently, the supernatant was removed, and the beads were washed for four times using PBS. The immunoprecipitated protein were removed from the beads by heating in 1× sample loading buffer at 100°C for 10 min, and the samples were subjected to Western blot analyses using anti-α-syn antibody (#2642; 1:1,000 dilution, Cell Signaling Technology).

### Plasmid Transfection

The plasmid DNA used here was a pcDNA3.1 vector containing a firefly luciferase gene (pcDNA3.1-luciferase) or a UBC9 gene (pcDNA3.1-UBC9). The plasmid DNA was propagated in *E. coli* (strain DH5α) and purified with a Qiagen Plasmid Mini Kit (Qiagen, Tokyo, Japan). The absorbance ratio of the plasmid DNA solution at wavelengths of 260 and 280 nm was measured to be between 1.8 and 2.0. SH-SY5Y cells were seeded on a six-well plate and cultured to 80% confluence. Lipofectamine 3,000 (Invitrogen, Carlsbad, CA, USA) was mixed with Opti-MEM medium (Gibco). Separately, P3000™ and pcDNA3.1-UBC9 were mixed with Opti-MEM medium for 10 min. The two mixed solutions were combined and incubated for 15 min at room temperature, then added to cells for 6 h of incubation. Subsequently, the complex medium was replaced with regular FBS-supplemented culture medium. After 48 h of incubation, the regular medium was changed to serum-free medium prior to METH exposure.

### RNA Interference and Transfection

SiSUMO-1, a small interfering RNA (siRNA) targeting SUMO-1, was synthesized by GenePharma (Suzhou, China). The sequences of siSUMO-1 were as follows: siSUMO-1#1 (human, 5′-GAGAAUUGCUGAUAAUCAUTT-3′) and siSUMO-1#2 (human, 5′-GACAGGGUGUUCCAAUGAATT-3′). The sequence of the control siRNA (siNC) was as follows: 5′-UUCUCCGAACGUGUCACGUTT-3′. SH-SY5Y cells were seeded on a six-well plate. When the cells reached 80% confluence, Lipofectamine 3,000 reagent and 100 nmol siRNA were mixed with Opti-MEM medium. The mixed solution was incubated for 15 min at room temperature and then added to the cells for another 4–6 h of incubation. Subsequently, the complex medium was replaced with regular FBS-supplemented culture medium. After 48 h of incubation, the regular medium was changed to non-serum medium prior to METH exposure.

### Virus Production and Establishment of Stable Expression in Cells

The synthesis of the lentiviruses was based on our recent studies (Qiao et al., 2014; Chen R. et al., 2016). In brief, the sequences of WT α-syn, and α-syn-2KR (K96R and K102R) were cloned into a pGag/Pol-LV vector and transfected into HEK293FT cells with pRev 1.0 and pVSV-G vectors. LV-WT-α-syn and LV-α-syn-2KR were harvested at 10^9^ transducing units per milliliter. LV-GFP was used as the control virus. These lentiviruses all conferred puromycin resistance. SH-SY5Y cells were plated in six-well plates and cultured in DMEM/F12 (1:1) containing FBS. The cells were exposed to a concentration range of puromycin to screen for the lowest concentration of puromycin that would kill all the SH-SY5Y cells. Then, SH-SY5Y cells were infected with LV-GFP, LV-WT-α-syn or LV-α-syn-2KR for 48 h. Subsequently, the cells treated with lentiviruses were treated with 2 μg/ml puromycin to screen for SH-SY5Y cells stably expressing WT α-syn, α-syn-2KR or GFP. Afterwards, these cells were plated in six-well plates and treated with METH for the next experiment.

### Viral Vector Production and Stereotaxic Injections

RAAV2 was used as a gene delivery vehicle for WT α-syn and α-syn-2KR. The viral vectors were propagated in HEK293T cells using the pDG2 helper plasmid. AAV2-WT α-syn and AAV2-α-syn-2KR were harvested with 10^10^ transducing units per milliliter. AAV2-EGFP was used as the control. The stereotaxic injection protocol was based on the studies from our lab (Chen R. et al., 2016; Xu et al., 2017). Healthy adult male C57 mice were divided randomly into five groups (*n* = 5/group): the AAV2-EGFP group, the AAV2-WT α-syn group, the AAV2-α-syn-2KR group, the AAV2-WT α-syn + METH group, and the AAV2-α-syn-2KR + METH group. The animals were anesthetized by i.p. injection of 1% pentobarbital before the surgical procedures. The anesthetized mice were fixed in a stereotaxic frame (Domitor, Wood Dale, IL, USA), and an incision was made on the skin overlying the skull. A 50 μl Hamilton syringe was used to inject 2 μl of AAV2-EGFP, AAV2-WT α-syn or AAV2-α-syn-2KR at a rate of 1 μl/min into the right striatum at the following stereotaxic coordinates: 0.38 mm rostral to Bregma, 1.78 mm lateral to the midline (right side), and 3.25 mm ventral to the dura, with the bite bar set at zero. After injection, the cannula remained *in situ* for an additional 1 min before being withdrawn slowly and gently. The striatum rather than the prefrontal cortex was selected to investigate the effect of mutations of lysine 96 and lysine 102 on α-syn SUMOylation *in vivo* because METH primarily targets dopaminergic neurons and the striatum has been shown to be the major target site for METH. The striatum also contains the highest density of dopaminergic synapses, whereas dopamine terminals in the prefrontal cortex are sparse in comparison (Volkow et al., 2001; Chang et al., 2005). After 2 days of recovery, the animals were treated with saline vehicle or METH as before.

### Statistical Analysis

All data are summarized as the mean ± standard deviation (SD) from at least three independent replicates. Statistical analysis was conducted by using one-way ANOVA followed by least significant difference (LSD) *post hoc* analysis or the Mann-Whitney U test for two independent samples (as appropriate) using the scientific statistical software SPSS version 19.0 (SPSS Inc., Chicago, IL, USA). A *p* value of <0.05 was considered statistically significant.

## Results

### METH Influences SUMO-1 and α-Syn Protein Expression *in vitro* and *in vivo*

**Figure 1:**
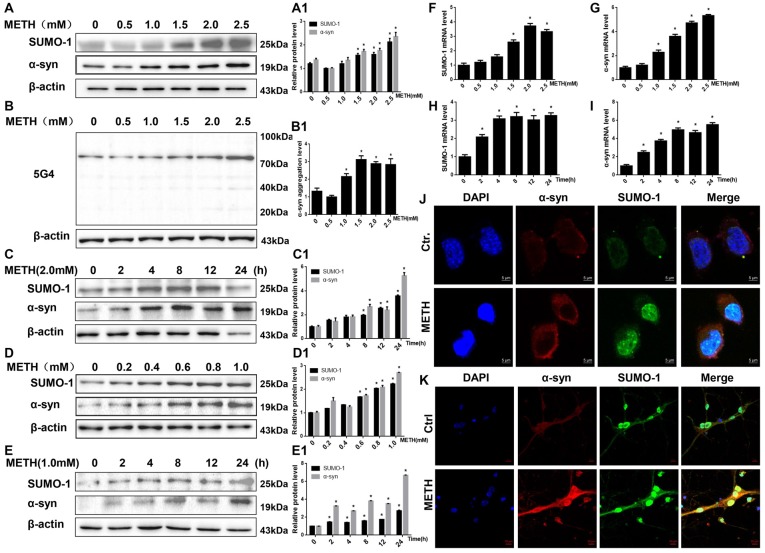
Methamphetamine (METH) increases small ubiquitin-related modifier 1 (SUMO-1) and alpha-synuclein (α-syn) protein expression *in vitro*. METH exposure up-regulates the protein expression of SUMO-1, α-syn and the aggregation of α-syn in a dose-dependent and time-dependent manner in SH-SY5Y cells and primary cultured neurons. SH-SY5Y cells were exposed to 0.5 mM-2.5 mM METH for 24 h **(A,B,A1,B1)** and 2.0 mM METH for 2–24 h **(C,C1)**. Primary cultured neurons were exposed to 0.2–1.0 mM METH for 24 h **(D,D1)** and 1.0 mM METH for 2–24 h **(E,E1)**. Western blot **(A–E)** and quantitative analyses **(A1–E1)** were performed to determine the levels of SUMO-1, α-syn and aggregated α-syn. RT-QPCR **(F–I)** was performed to determine SUMO-1 and α-syn mRNA expression in SH-SY5Y cells. SUMO-1 and α-syn were expressed at higher levels in METH-treated SH-SY5Y cells (2.0 mM, 24 h) and primary cultured neurons (1.0 mM, 24 h) than in the control group, according to analysis with a fluorescence microscope **(J,K)**. SUMO-1 was stained with anti-SUMO-1 antibody (green); α-syn was stained with anti-α-syn antibody (red); nuclei were counterstained with DAPI (blue). β-Actin was used as a loading control. *p < 0.05 compared with the control group. The data shown in **(A–I)** were analyzed using one-way ANOVA followed by least significant difference (LSD) *post hoc* analyses.

We treated SH-SY5Y cells and primary cultured neurons with a range of METH doses for 24 h or treated with 2.0 mM or 1.0 mM METH for 2–24 h to determine whether METH affects the expression of the SUMO-1 and α-syn proteins. According to the Western blot results, METH increased the expression of the SUMO-1 and α-syn proteins in a dose-dependent (Figures 1A,D) and time-dependent (Figures 1C,E) manner. For example, after 24 h of exposure, the expression of the SUMO-1 protein was significantly increased by 1.77-fold in the METH-treated (2.5 mM) SH-SY5Y cells (*n* = 3, *p < 0.05). Similarly, the expression of the α-syn protein was markedly increased by 1.73-fold in SH-SY5Y cells treated with 2.5 mM METH for 24 h (*n* = 3, *p < 0.05). Level of aggregated α-syn was also increased by 1.76-fold in a dose-dependent manner following METH exposure (2.5 mM, 24 h; Figure 1B, *p < 0.05). In addition, after a 24-h exposure, levels of the SUMO-1 protein were significantly increased by 2.23-fold in the METH-treated (1.0 mM) primary cultured neurons (*n* = 3, *p < 0.05). Likewise, α-syn protein expression was observably increased by 2.71-fold in primary cultured neurons treated with 1.0 mM METH for 24 h (*n* = 3, *p < 0.05).

The RT-QPCR results showed that in SH-SY5Y cells METH increased SUMO-1 and α-syn mRNA levels in a dose-dependent (Figures 1F,G, *p < 0.05) and time-dependent (Figures 1H,I, *p < 0.05) manner. Immunofluorescence results also show that levels of the SUMO-1 and α-syn proteins were increased in the METH-treated SH-SY5Y cells (2.0 mM, 24 h) and primary cultured neurons (1.0 mM, 24 h; Figures 1J,K). However, levels of the SUMO-1 and α-syn proteins were not significantly altered in SH-SY5Y cells treated with a low dose of METH (0.5 mM and 1.0 mM) or primary cultured neurons (0.2 mM and 0.4 mM) after a 24-h exposure (*n* = 3, *p* > 0.05). Therefore, increased SUMO-1 and α-syn protein expression caused by high doses of METH may partly lead to high-dose METH-induced neurotoxicity. However, it indicates that SUMO-1 and α-syn do not appear to be involved in low-dose METH-induced neurotoxicity.

**Figure 2:**
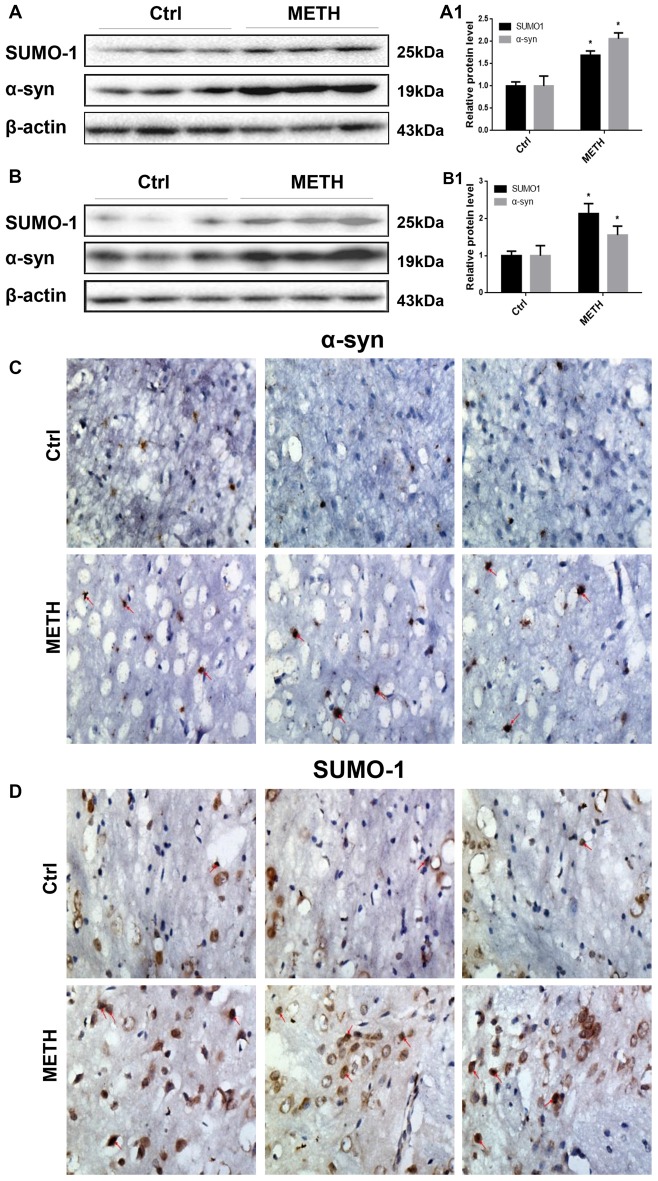
METH increases SUMO-1 and α-syn protein expression *in vivo*. METH increases SUMO-1 and α-syn expression in the prefrontal cortex **(A,A1)** and striatum **(B,B1)** of male C57 BL/6 mice. Male C57 mice were randomly divided into a control group and a subacute group. Western blot **(A,B)** and quantitative analyses **(A1,B1)** were performed to determine SUMO-1 and α-syn protein expression in the prefrontal cortex and striatum. Immunostaining of the mouse striatum also showed that SUMO-1 and α-syn expression were increased after METH exposure **(C,D)**. β-Actin was used as a loading control. *p < 0.05 compared with the control group. The data shown in **(A,B)** were analyzed using the Mann-Whitney U test.

To provide evidence for α-syn SUMOylation in the brain, we determined SUMO-1 and α-syn protein expression in the prefrontal cortex and striatum of mice following subacute METH exposure. In the Western blot results obtained from the prefrontal cortex, the SUMO-1 protein expression was 1.69-fold higher in the subacute exposure groups than in the control group (Figure 2A, *p < 0.05). The α-syn protein level was increased 2.06-fold in the subacute exposure groups compared with that in the control group (Figure 2A, *p < 0.05). Similarly, subacute METH exposure increased SUMO-1 and α-syn protein expression (2.14-fold and 1.56-fold, respectively) in the striatum (Figure 2B, *p < 0.05). Immunohistochemistry was used to examine the levels of SUMO-1 and α-syn in the mouse striatum following subacute METH exposure. It was observed that METH-exposed mice exhibited increased SUMO-1 and α-syn immunoreactivity in the striatum (Figures 2C,D). Thus, subacute METH exposure increases SUMO-1 and α-syn protein expression *in vivo*. The results *in vivo* are consistent with the data *in vitro*.

### METH Decreases the SUMOylation Level of α-Syn via UBC9

**Figure 3:**
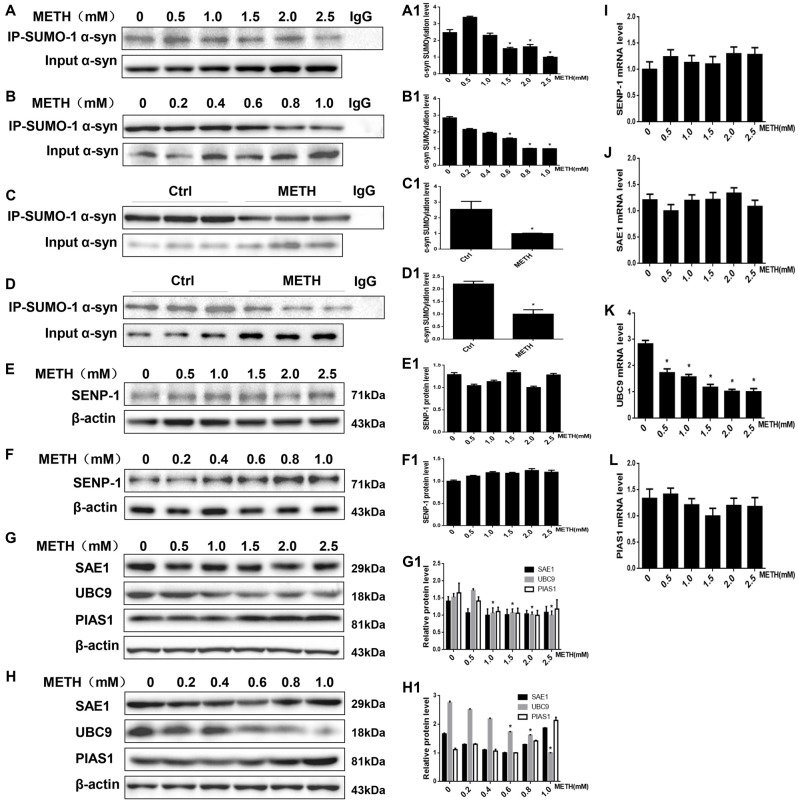
METH decreases the level of SUMOylated α-syn via UBC9. Co-immunoprecipitation results show that METH decreases the level of SUMOylated α-syn *in vitro* and *in vivo*. The unique E2-conjugating enzyme, UBC9, is a key factor that is also reduced in METH-induced cells. SH-SY5Y cells **(A,A1)** and primary cultured neurons **(B,B1)** were exposed to a range of METH doses for 24 h. Male C57 mice were divided randomly into a control group and a subacute group. Cells **(A,B,A1,B1)** and tissues **(C,D,C1,D1)** were immunoprecipitated with an anti-SUMO-1 antibody, followed by western blot with an anti-α-syn antibody. Cells and tissues immunoprecipitated with IgG were used as a negative control. Levels of several enzymes involved in the SUMOylation cycle were measured in SH-SY5Y cells **(E,G,E1,G1)** and primary cultured neurons **(F,H,F1,H1)**. Western blot **(A–H)** and quantitative analyses **(A1–H1)** were performed to determine the level of SUMOylated α-syn. RT-QPCR **(I–L)** was performed to determine several enzymes mRNA expression in SH-SY5Y cells. β-Actin was used as a loading control. *p < 0.05 compared with the control group. The data shown in **(A,B,E–L)** were analyzed using one-way ANOVA followed by LSD *post hoc* analyses, whereas the data shown in **(C,D)** were analyzed using the Mann-Whitney U test.

The results presented above suggest that METH exposure up-regulates SUMO-1 and α-syn protein expression *in vitro* and *in vivo*. However, based on the co-immunoprecipitation results, the SUMOylation level of α-syn was decreased by relatively higher doses of METH *in vitro* (Figures 3A,B). The SUMOylation level of α-syn was decreased by 59.6% and 64.9% in METH-treated SH-SY5Y cells (2.0 mM, 24 h) and primary cultured neurons (1.0 mM, 24 h; *n* = 3, *p < 0.05). The decrease in α-syn SUMOylation was also involved in METH-induced neurotoxicity *in vivo* (Figures 3C,D). The SUMOylation level of α-syn was reduced by 60.7% and 54.6% in the mouse prefrontal cortex and striatum, respectively, following subacute METH exposure (*n* = 3, *p < 0.05). Therefore, the inhibition of α-syn SUMOylation may partially contribute to METH-induced neurotoxicity.

SUMOylation is fundamental in eukaryotic cells, and knockdown or deletion of UBC9, a pivotal constituent of the SUMOylation pathway, is lethal in mammalian cells (Gupta et al., 2014). The balance between UBC9-mediated conjugation and SENP-mediated deconjugation determines the SUMOylation state of specific protein, such as α-syn, and the mechanisms underlying the regulation of these processes are a very active area of research (Wilkinson et al., 2010). We measured the levels of enzymes involved in the SUMOylation cycle to identify the mechanism by which METH regulates the level of α-syn SUMOylation. The expression of the SENP1 protein was not markedly altered in SH-SY5Y cells or primary cultured neurons (Figures 3E,F, *p* > 0.05). Compared with the control group, the UBC9 expression level was reduced in SH-SY5Y cells and primary cultured neurons treated with a range of METH doses for 24 h (*n* = 3, *p < 0.05), whereas the levels of two SUMOylation factors the E1 ligase SAE1 and the E3 ligase PIAS1 showed no obvious changes (Figures 3G,H, *p* > 0.05). The RT-QPCR results also showed that in SH-SY5Y cells METH decreased UBC9 mRNA level in a dose-dependent manner (Figure 3K, *p < 0.05), whereas the mRNA levels of SENP-1, SAE1 and PIAS1 all showed no obvious changes (Figures 3H–J, *p* > 0.05). Therefore, we hypothesize that among the SUMOylation enzymes, the unique E2-conjugating enzyme, UBC9, is a key element that regulates the level of SUMOylated α-syn in METH-treated cells. The mechanism by which METH induces α-syn SUMOylation is mediated by UBC9.

### The Level of SUMOylated α-Syn in METH-Treated Cells Is Increased When UBC9 Is Overexpressed

**Figure 4:**
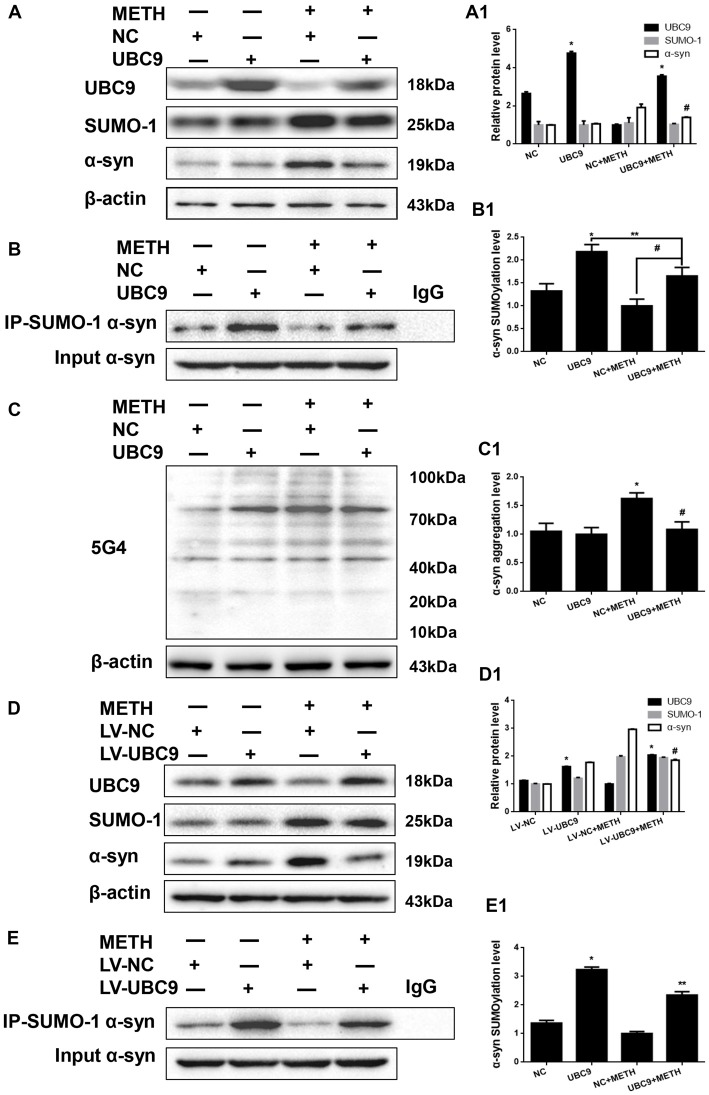
The level of SUMOylated α-syn in METH-treated cells increases when UBC9 is overexpressed. SH-SY5Y cells **(A–C,A1–C1)** and primary cultured neurons **(D,E,D1,E1)** were transfected with pcDNA3.1-NC or pcDNA3.1-UBC9 and LV-NC or LV-UBC9, respectively. Cells were treated with or without METH. Then, the cells were immunoprecipitated with an anti-SUMO-1 antibody, followed by western blot with an anti-α-syn antibody. Cells immunoprecipitated with IgG were used as a negative control. Western blot **(A–E)** and quantitative analyses **(A1–E1)** were performed to evaluate the efficiency of UBC9 overexpression, the expression of SUMO-1, and the expression, aggregation and SUMOylation of α-syn. β-Actin was used as a loading control. *p < 0.05 compared with the NC group. **p < 0.05 compared with the LV-UBC group. ^#^*p* < 0.05 compared with the METH-treated NC group. The data were analyzed using one-way ANOVA followed by LSD *post hoc* analyses.

To test how cellular SUMOylation of α-syn relies on the catalytic activity of UBC9, we effectively overexpressed UBC9 using a plasmid in SH-SY5Y cells. The level of the UBC9 protein increased significantly by 1.80-fold compared with the level in the negative control group (Figure 4A, *p < 0.05). As expected in Figure 4B, the level of SUMOylated α-syn was increased by 1.64-fold in cells transfected with the UBC9 plasmid compared with the level in negative control group (*p < 0.05), and the level of SUMOylated α-syn was increased by 1.65-fold in METH-treated cells transfected with the UBC9 plasmid compared with the level in METH-treated group (^#^*p* < 0.05), although the level of SUMOylated α-syn in METH-treated cells transfected with the UBC9 plasmid was reduced by 24.2% compared with the level in UBC9-transfected group (**p < 0.05). More importantly, METH exposure significantly increased the expression of α-syn protein; this increase was mitigated by 27.2% after transfection with the UBC9 plasmid (Figure 4A, ^#^*p* < 0.05). In METH-treated cells transfected with the UBC9 plasmid, the aggregation of α-syn was also mitigated by 33.1% compared with the group treated with METH alone (Figure 4C, ^#^*p* < 0.05).

We verified the results obtained in SH-SY5Y cells by overexpressing the UBC9 gene in primary cultured neurons using LV-UBC9. The level of the UBC9 protein expression increased by 1.44-fold, and the level of SUMOylated α-syn was increased by 2.37-fold (Figures 4D,E, *p < 0.05), although the level of SUMOylated α-syn in METH-treated cells with the LV-UBC9 was also reduced by 27.5% compared with the level in LV-UBC9 group (**p < 0.05). Similarly, the increase in α-syn protein expression in response to METH was diminished by 37.4% after exposure to LV-UBC9 (Figure 4D, ^#^*p* < 0.05), the SUMOylation level of α-syn in response to METH was increased by 1.69-fold after exposure to LV-UBC9 (Figure 4E, ^#^*p* < 0.05). Based on these results, UBC9 is an upstream regulator of α-syn SUMOylation, and the increase in α-syn SUMOylation plays a critical role in relieving METH-induced α-syn aggregation.

### Silencing of SUMO-1 Expression Exacerbates α-Syn Aggregation in METH-Treated Cells

**Figure 5:**
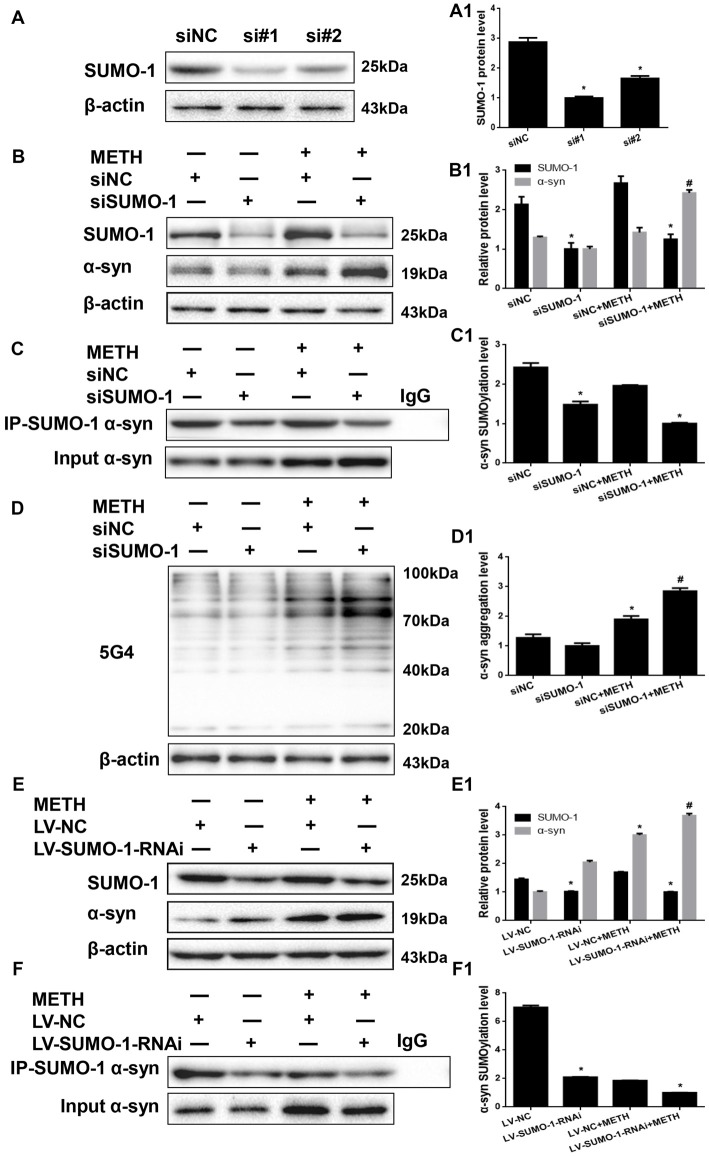
Silencing of SUMO-1 expression exacerbates α-syn aggregation in METH-treated cells. First, SH-SY5Y cells were transfected with siNC, siSUMO-1#1 or siSUMO-1#2 **(A)**. Then, SH-SY5Y cells **(B–D,B1–D1)** and primary cultured neurons **(E,F,E1,F1)** were transfected with siNC or siSUMO-1 and LV-NC or LV-SUMO-1-RNAi, respectively and the cells were treated with or without METH. The cells were immunoprecipitated with an anti-SUMO-1 antibody, followed by western blot with an anti-α-syn antibody. Cells were immunoprecipitated with IgG were used as a negative control. Western blot **(A–F)** and quantitative analyses **(A1–F1)** were performed to evaluate the expression of SUMO-1 and the expression, aggregation and SUMOylation of α-syn. β-Actin was used as a loading control. *p < 0.05 compared with the NC group. ^#^*p* < 0.05 compared with the METH-treated NC group. The data were analyzed using one-way ANOVA followed by LSD *post hoc* analyses.

We investigated whether SUMO-1 knockdown affects METH-induced α-syn aggregation *in vitro* to further assess the role of SUMO-1 in METH-induced α-syn aggregation. First, we respectively transfected each of the two siRNA sequences (siSUMO-1 #1 and #2, 100 nM) or control siRNA (siNC; 100 nM) into SH-SY5Y cells for 48 h. SUMO-1 expression was markedly reduced by 65.2% and 42.5% in cells transfected with siSUMO-1 #1 and #2 (Figure 5A, *p < 0.05). Subsequently, we selected siSUMO-1 #1 as the siSUMO-1 for the next experiment because it achieved a better silencing than #2. Then, we transfected siSUMO-1 or siNC (100 nM) into SH-SY5Y cells followed by METH exposure (2.0 mM, 24 h). Western blot analyses showed that silencing of SUMO-1 expression decreased the level of SUMOylated α-syn by 38.8% (Figure 5C, *p < 0.05). In addition, METH exposure increased the expression and aggregation of α-syn protein, and this effect was significantly exacerbated by 1.70-fold (Figure 5B, *p < 0.05) and 1.50-fold (Figure 5D, *p < 0.05) upon SUMO-1 silencing.

To verify the results obtained from SH-SY5Y cells, we also infected primary cultured neurons with LV-SUMO-1-RNAi or control LV-GFP (100 nM) followed by METH exposure (1.0 mM, 24 h). The level of SUMOylated α-syn decreased by 70.1% in primary cultured neurons infected with lentivirus LV-SUMO-1-RNAi (Figure 5F, *p < 0.05). Notably, α-syn expression was increased by 1.23-fold after co-exposure to METH and LV-SUMO-1-RNAi compared with the level in the METH group (Figure 5E, ^#^*p* < 0.05). Thus, silencing SUMO-1 reduced the level of α-syn SUMOylation and exacerbated α-syn overexpression and aggregation induced by METH *in vitro*.

### Impaired SUMOylation of α-Syn Accelerates α-Syn Aggregation Induced by METH in SH-SY5Y Cells

**Figure 6:**
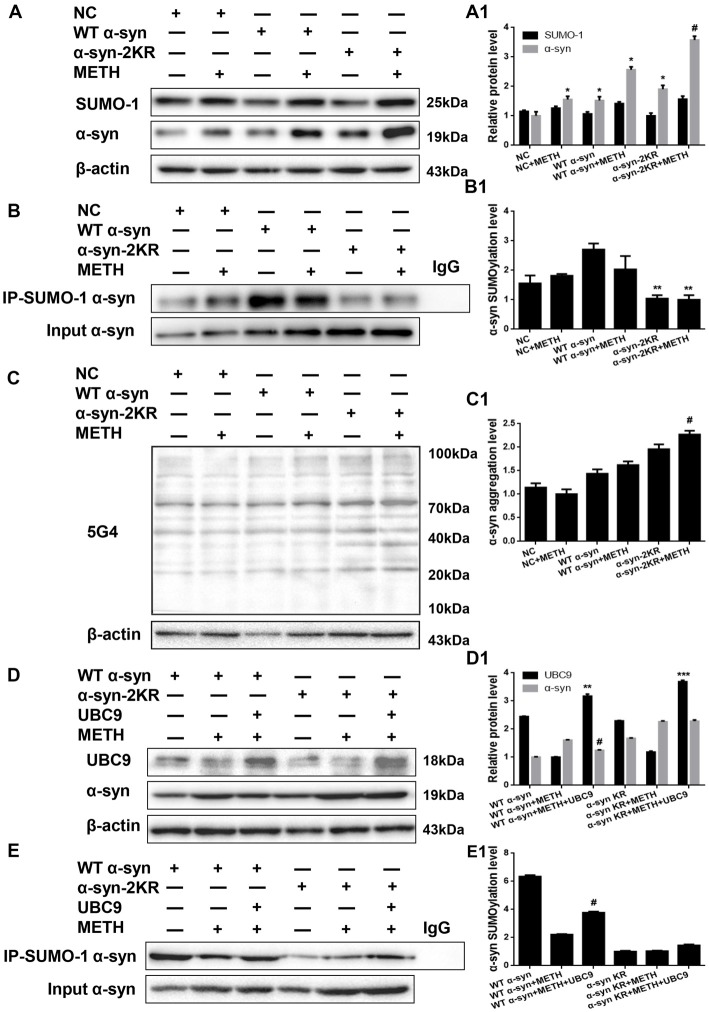
Impaired SUMOylation of α-syn accelerates α-syn aggregation induced by METH in SH-SY5Y cells. SH-SY5Y cells stably expressing GFP, WT α-syn or α-syn-2KR were treated with or without 2.0 mM METH for 24 h. **(A–C,A1–C1)** In addition, UBC9 was overexpressed in SH-SY5Y cells stably expressing WT α-syn and α-syn-2KR **(D,E,D1,E1)**. Western blot **(A–E)** and quantitative analyses **(A1–E1)** were performed to evaluate the efficiency of α-syn expression, and the expression of SUMO-1 and UBC9, and the expression, aggregation and SUMOylation of α-syn. β-Actin was used as a loading control. *p < 0.05 compared with the LV-GFP group. **p < 0.05 compared with SH-SY5Y cells stably expressing WT α-syn. ***p < 0.05 compared with SH-SY5Y cells stably expressing α-syn-2KR. ^#^*p* < 0.05 vs. METH-treated SH-SY5Y cells stably expressing WT α-syn. The data were analyzed using one-way ANOVA followed by LSD *post hoc* analyses.

α-Syn has multiple SUMOylation acceptor sites, the most crucial of which are lysine 96 and lysine 102 (Shahpasandzadeh et al., 2014). To further evaluate the effect of α-syn SUMOylation, we compared the aggregation propensity and neurotoxicity of α-syn with K96R and K102R mutations and WT α-syn. For this purpose, we screened for SH-SY5Y cells stably expressing WT α-syn, α-syn-2KR or GFP and treated those cells with METH. Western blot analyses showed higher α-syn expression levels in SH-SY5Y cells stably expressing WT α-syn (1.52-fold) and α-syn-2KR (1.91-fold) than in the negative control group (Figure 6A, *p < 0.05). Although SUMO-1 expression did not differ significantly in these cells (*p* > 0.05), SUMOylation of α-syn was impaired by 61.6% in SH-SY5Y cells stably expressing α-syn-2KR compared with that in SH-SY5Y cells stably expressing WT α-syn (Figure 6B, **p < 0.05). Furthermore, METH exposure significantly increased the expression and aggregation of α-syn, and these increases were accelerated by 1.39-fold and 1.25-fold, respectively, when SUMOylation of α-syn was impaired (Figures 6A,C, ^#^*p* < 0.05).

We effectively overexpressed the UBC9 gene in SH-SY5Y cells stably expressing WT α-syn or α-syn-2KR to confirm the results presented above. UBC9 expression was increased by 1.30-fold and 1.61-fold in SH-SY5Y cells stably expressing WT α-syn (**p < 0.05) and α-syn-2KR (***p < 0.05) along with UBC9 plasmid (Figure 6D). As expected, compared with the level in SH-SY5Y cells stably expressing WT α-syn treated with METH, the α-syn expression level was reduced by 22.7% and the level of SUMOylated α-syn was increased by 1.71-fold in cells transfected with the UBC9 plasmid (Figures 6D,E, ^#^*p* < 0.05). In contrast, compared with the level in SH-SY5Y cells stably expressing α-syn-2KR that were treated with METH, the α-syn expression level and the level of SUMOylated α-syn were not significantly different in cells transfected with the UBC9 plasmid (Figures 6D,E, *p* > 0.05). These results further verify the finding that impaired SUMOylation of α-syn accelerates α-syn aggregation in METH-treated cells. In addition, UBC9 is a key factor that regulates the level of α-syn SUMOylation in SH-SY5Y cells stably expressing WT α-syn, but not in SH-SY5Y cells stably expressing α-syn-2KR.

### Impaired SUMOylation of α-Syn Hinders α-Syn Degradation Through the UPS and the ALP

**Figure 7:**
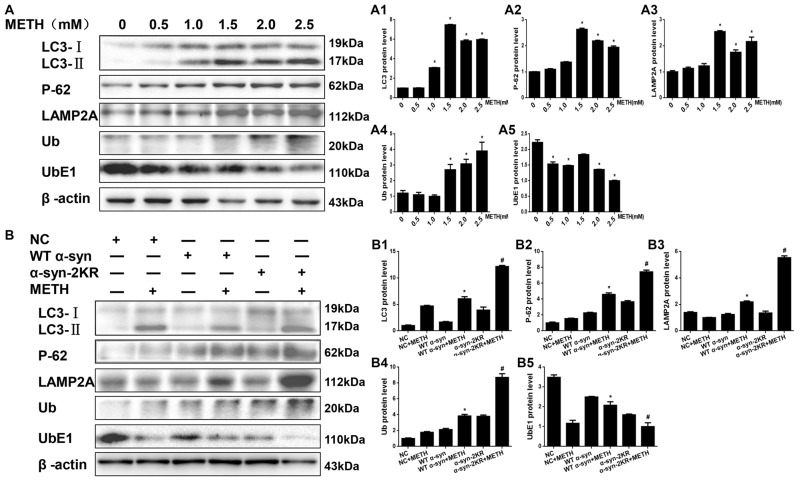
Impaired SUMOylation of α-syn hinders α-syn degradation through the ubiquitin-proteasome system (UPS) and the autophagy-lysosome pathway (ALP). SH-SY5Y cells were exposed to METH in a dose-dependent manner for 24 h **(A,A1–A5)**. SH-SY5Y cells stably expressing GFP, WT α-syn or α-syn-2KR were treated with or without 2.0 mM METH for 24 h **(B,B1–B5)**. Western blot **(A,B)** and quantitative analyses **(A1–A5,B1–B5)** were performed to evaluate the expression of UbE1 and Ub, which are involved in the UPS, and of LC3-II, P-62 and Lysosomal associated membrane protein-2 (LAMP-2), which are involved in the ALP. β-Actin was used as a loading control. *p < 0.05 compared with the control or LV-GFP group. ^#^*p* < 0.05 compared with the METH-treated SH-SY5Y cells stably expressing WT α-syn. Data were analyzed using one-way ANOVA followed by LSD *post hoc* analyses.

After defining the establishing that impaired SUMOylation of α-syn accelerated α-syn aggregation, we sought to explore how the SUMOylation of α-syn influenced its aggregation *in vitro*. Therefore, we tested the expression of UbE1 and Ub, which are involved in the UPS, and LC3-II, P-62 and LAMP2A, which are involved in the ALP. According to the Western blot results, METH increased Ub, LC3-II, P-62 and LAMP2A protein expression and decreased the level of the UbE1 protein in SH-SY5Y cells in a dose-dependent manner (Figure 7A, *p < 0.05). These results indicated METH reduced UbE1 activity and induced Ub accumulation, and the ratio of UbE1 to Ub was also reduced. Moreover, METH impaired autophagic flux and blocked the fusion of autophagosomes and lysosomes. The METH-induced decrease in UbE1 protein expression and the UbE1/Ub protein expression ratio in SH-SY5Y cells stably expressing α-syn-2KR were aggravated by 45.3% and 74.5%, respectively, compared with the values in SH-SY5Y cells stably expressing WT α-syn (Figure 7B, ^#^*p* < 0.05). In addition, the METH-induced increases in LC3-II, P-62 and LAMP2A protein expression in SH-SY5Y cells stably expressing α-syn-2KR were also aggravated by 2.01-fold, 1.62-fold and 2.53-fold, respectively, compared with the values in METH-treated SH-SY5Y cells stably expressing WT α-syn (Figure 7B, ^#^*p* < 0.05). Based on these results, impaired SUMOylation of α-syn hinders α-syn degradation through the UPS and the ALP following METH exposure.

### Mutations in the Major SUMOylation Acceptor Sites of α-Syn Exacerbate METH-Induced α-Syn Aggregation *in vivo*

**Figure 8:**
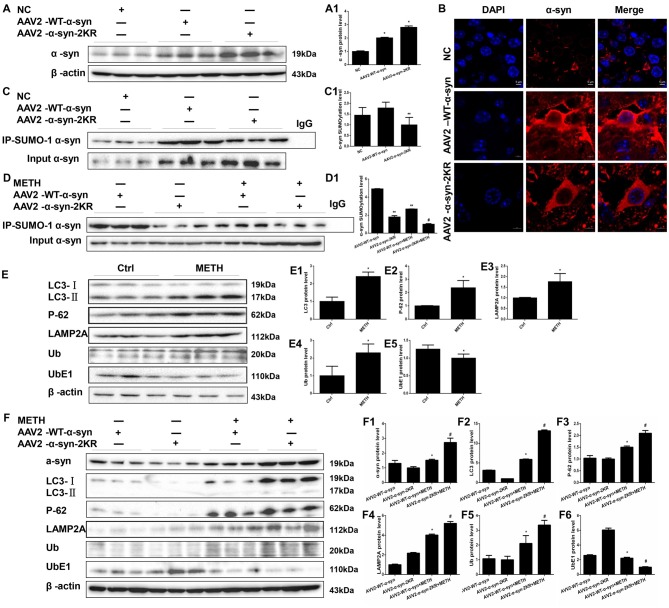
Mutations in the major SUMOylation acceptor sites of α-syn exacerbate METH-induced α-syn aggregation *in vivo*. Adenovirus were injected into the right striatum of mice using a standard stereotaxic positioning system. After 2 days of recovery, mice received saline or METH by i.p. injection. Striatal tissues (right side) were harvested at 24 h after the injection of the last dose. The tissues were immunoprecipitated with an anti-SUMO-1 antibody, followed by western blot with an anti-α-syn antibody. Tissues immunoprecipitated with IgG were used as a negative control. Western blot **(A,C)** and quantitative analyses **(A1,C1)** were performed to evaluate the efficiency of WT α-syn and α-syn-KR expression. Immunofluorescence staining of mouse striatum sections showed that α-syn expression increased after injection of adenovirus **(B)**. Western blot **(D–F)** and quantitative analyses **(D1,E1–5,F1–6)** were also performed to the levels of the SUMO-1, UbE1, Ub, LC3-II, P-62 and lysosomal associated membrane protein 2A (LAMP2A) proteins and the expression of SUMOylated α-syn. β-Actin was used as a loading control. *p < 0.05 compared with the AAV2-NC or control group. **p < 0.05 compared with the AAV2-α-syn group. ^#^*p* < 0.05 compared with the AAV2-α-syn + METH-treated group. The data shown in **(A,C,D,F)** were analyzed using one-way ANOVA followed by LSD *post hoc* analyses, whereas the data shown in **(E)** were analyzed using the Mann-Whitney U test.

AAV2-EGFP, AAV2-WT α-syn and AAV2-α-syn-2KR were injected into the right striatum of mice using a standard stereotaxic positioning system and then mice were treated with or without METH to further verify that the mutations at lysines 96 and 102 exacerbate METH-induced α-syn aggregation *in vivo*. As shown in Supplementary Figure S1, immunofluorescence staining shows that the adenovirus had been successfully infected into striatum. According to the Western blot results, striatal levels of the α-syn protein in mice treated with AAV2-WT-α-syn and AAV2-α-syn-2KR were observably increased by 2.02-fold and 2.81-fold, respectively, compared with the expression in the negative control (Figure 8A, *p < 0.05). The immunofluorescent staining showed the same results (Figure 8B). The striatal level of SUMOylated α-syn was impaired by 44.2% in the AAV2-α-syn-2KR group compared with the AAV2-WT-α-syn group (Figure 8C, **p < 0.05), and the striatal level of SUMOylated α-syn was decreased by 62.8% in the METH-treated AAV2-α-syn-2KR group compared with the METH-treated AAV2-WT-α-syn group (Figure 8D, ^#^*p* < 0.05). Consistent with results from the *in vitro* experiments, METH exposure significantly increased the expression of α-syn, and this increase was intensified by 1.79-fold when lysines 96 and 102 of α-syn were mutated (Figure 8F, ^#^*p* < 0.05). Subacute METH exposures decreased UbE1 protein expression and increased Ub, LC3-II, P-62 and LAMP2A protein expression in the striatum of mice (Figure 8E, *p < 0.05). The METH-induced decreases in UbE1 and UbE1/Ub protein expression in mice injected with AAV2-α-syn-2KR were aggravated by 55.0% and 71.7%, respectively, compared with those in mice injected with AAV2-WT-α-syn (Figure 8F, ^#^*p* < 0.05). In addition, METH exposure also aggravated the increases in LC3-II, P-62 and LAMP2A protein expression in mice injected with AAV2-α-syn-2KR by 2.22-fold, 1.40-fold and 1.30-fold, respectively compared with the values in mice injected with AAV2-WT-α-syn (Figure 8F, ^#^*p* < 0.05). These results indicate that SUMOylation-resistant α-syn (α-syn-2KR) leads to an increased proportion of α-syn aggregation upon METH exposure. Moreover, the UPS and ALP are related to the SUMOylation of α-syn, thus influencing α-syn aggregation induced by METH *in vivo*. The results obtained from *in vivo* studies showing that mutations of lysines 96 and 102 exacerbate METH-induced α-syn aggregation further confirm the results obtained *in vitro*.

## Conclusion

In the present study, we report that SUMO-1 and α-syn expression are increased after high-dose METH exposure *in vitro* and *in vivo*. However, the level of SUMOylated α-syn is decreased by METH *in vitro* and *in vivo*. The blockade of α-syn SUMOylation may contribute to METH-induced α-syn aggregation. One possible reason is that UBC9, a key factor that regulates the SUMOylation level of α-syn, is reduced in METH-induced cells. If UBC9 is overexpressed, the SUMOylation level of α-syn is increased remarkably, and the increase in α-syn SUMOylation plays a critical role in relieving α-syn overexpression and aggregation induced by METH. Conversely, the SUMOylation level of α-syn is decreased when SUMO-1 expression is blocked. The overexpression and aggregation of α-syn induced by METH is aggravated by the reduction of α-syn SUMOylation. Furthermore, mutations in the major SUMOylation acceptor sites of α-syn block SUMOylation of α-syn and exacerbate METH-induced α-syn overexpression and aggregation *in vitro* and *in vivo*. Additionally, SUMOylation of α-syn influence α-syn aggregation and degradation through the UPS and the ALP. These findings together with previous studies indicate that SUMOylation has a protective effect not only against several neurodegenerative diseases (Vijayakumaran et al., 2015), but also against α-syn overexpression and aggregation induced by METH.

α-Syn is an abundant 140-residue neuronal protein, that is principally located in neuronal presynaptic terminals under physiological conditions, close to synaptic vesicles. Deposits of α-syn have been identified in pathological aggregates, such as LBs and oligodendroglial inclusions, in patients with neurodegenerative disorders (Lee and Lee, 2002; Kahle, 2008; Vekrellis et al., 2011). Recent reports have demonstrated that METH exposure can lead to PD-like LBs in the substantia nigra and striatum of rats (Fornai et al., 2004; Lazzeri et al., 2007), and the major ingredient of LBs is a-syn fibrillization. Moreover, a-syn expression is increased in METH-treated neurons (Ajjimaporn et al., 2007; Chen et al., 2013). Aggregated α-syn contains various recruited factors, including Ub, SUMO, protein chaperones and proteasome components. In this study, we verify that METH induces α-syn overexpression and aggregation *in vitro* and *in vivo*, consistent with previous research. In addition, we propose the hypothesis that SUMOylation of α-syn plays a role in METH-induced α-syn overexpression and aggregation.

SUMOylation displays resemblances to ubiquitination in both the structure and the biochemistry of their conjugation. In contrast to ubiquitination, which mainly tags proteins for the UPS, SUMOylation has an amount of functional consequences for the target proteins. SUMOylation regulates protein interactions, affects their subcellular localization, and influences cell stress responses. Several recent studies have charactered increased SUMOylation as a neuroprotective factor in some cases. For instance, a study *in vitro* has explored the direct effect of SUMOylation on the aggregation susceptibility of α-syn. The study reveals that SUMOylation of a small portion of α-syn is sufficient to suppress its aggregation (Abeywardana and Pratt, 2015). Consistent with the experiment, *in vitro* study has revealed that a deficiency in the SUMOylation of α-syn exacerbates aggregation and causes detrimental increases in cellular toxicity (Krumova et al., 2011). While other studies come to almost completely opposite conclusions, SUMOylation facilitates α-syn aggregation by blocking its ubiquitin-dependent degradation pathways and promoting its accumulation (Rott et al., 2017). Additionally, SUMO labeling of LBs in tissues from patients with PD was reported by Kim et al. (2011), who also show that SUMO is recruited to α-syn inclusions induced by proteasome inhibition *in vitro*. However, the definite part of SUMO-1 in nervous system remains indistinct, and studies examining the relationship between SUMOylation and METH are not available. Therefore, for the first time, we investigate the potential direct or indirect roles of SUMO-1 in METH-induced aggregation of misfolded α-syn *in vitro* and *in vivo*. Our results show that METH reduces the level of SUMOylated α-syn, and METH-induced α-syn overexpression and aggregation are exacerbated by the reduction of α-syn SUMOylation level. It is said that the depletion of endogenous SUMO-1 sensitized the cells to oxygen/glucose deprivation or restoration of oxygen/glucose (Lee et al., 2009). And not only has that, silencing of SUMO-1 aggravates the toxicity of METH in our study. SUMOylation of α-syn is decreased not only by the silencing of SUMO-1 expression but also by mutations in the major SUMOylation acceptor sites of α-syn, and mutations decrease α-syn SUMOylation level specifically.

Notably, the level of SUMOylated α-syn was reduced by METH exposure in this study; the decrease may be due to UBC9, the unique E2-conjugating enzyme in the SUMOylation circle. Previous studies have shown that UBC9 level is correlated with SUMOylation level in hibernating squirrels and SH-SY5Y cells, and animals with higher SUMO conjugation levels are more bearable to ischemic insult (Lee et al., 2007, 2009, 2014). In one study, Lee et al. (2011) modeled several lines of UBC9 transgenic mice whose UBC9 expression was elevated generally to various degrees. These transgenic mice were found be observably more resistant to permanent middle cerebral artery occlusion (pMCAO), an animal stroke model, than corresponding wild-type animals. Higher UBC9 level in the brain resulted in lower infarction volumes under pMCAO (Lee et al., 2011). However, down-regulated SUMOylation by high concentration of UBC9 occurs *in vivo* as well (Wang et al., 2010). In our study, we overexpressed UBC9 protein because METH decreased the UBC9 level. As expected, the level of SUMOylated α-syn was increased upon UBC9 overexpression. UBC9 may be an upstream regulator of α-syn SUMOylation. The mechanism by which METH induces α-syn SUMOylation is mediated by UBC9. The METH-induced overexpression and aggregation of α-syn were mitigated in SH-SY5Y cells and primary cultured neurons transfected with the UBC9 plasmid. However, UBC9 did not produce this effect in cells containing mutations in the major SUMOylation acceptor sites of α-syn. Therefore, SUMOylation of α-syn is vital to maintain the normal structure of α-syn.

Intracellular mechanisms for the clearance of aberrantly folded proteins such as α-syn include two proteolytic pathways: the UPS and the ALP, both of which have potential roles in SUMO-1 modification. Decreases in UbE1 and UbE1/Ub and increases in LC3-II, P-62 and LAMP2A protein expression show that METH indeed impairs both the UPS and the ALP and thereby induces neurotoxicity. SUMO-1 has been identified to co-localize with lysosomes in α-syn aggregate-bearing cells under proteasome inhibition explicitly (Wong et al., 2013). On the one hand, there are several studies indicating that SUMOylated α-syn is mainly attached to the autophagy pathway, and non-SUMOylated α-syn primarily to the proteasome. Inhibition of α-syn SUMOylation gives rise to inefficient autophagy-mediated aggregate clearance and directs the protein to the proteasome (Shahpasandzadeh et al., 2014). On the other hand, downregulation of SUMOylation also hindered the clearance of β-syn by the 26S proteasome significantly and enhanced protein stability (Popova et al., 2018). Although the UPS and the ALP were previously reported to operate respectively, recent studies manifested the existence of specific mechanisms for selective collaboration between the UPS and the ALP, in particular in the event of toxic accumulation of protein aggregates. SUMO has also been found to associate with numerous key players in the UPS, such as Parkin, TRAF6 and CHIP (Um and Chung, 2006; Yan et al., 2010; Paul and Kumar, 2011). In this study, we preliminarily show that impaired SUMOylation of α-syn influences α-syn aggregation and degradation through the UPS and the ALP following METH exposure. SUMOylation of α-syn directly influences α-syn degradation through the ALP, whereas the influence of α-syn SUMOylation on α-syn degradation through the UPS may be modulated indirectly through other key players. However, the specific mechanism by which METH induces the degradation of SUMOylated α-syn through the UPS and the ALP requires further research.

In summary, we provide direct and indirect evidence from *in vitro* and *in vivo* studies showing SUMOylation contributes to relieve METH-induced α-syn aggregation. We characterize SUMOylation of α-syn as alleviating α-syn aggregation with METH exposure. UBC9 as the unique E2-conjugating enzyme is a key factor that modulates the level of SUMOylated α-syn in METH-exposed cells. The mechanism by which METH induces α-syn SUMOylation is mediated by UBC9. Meanwhile, the reduction in α-syn SUMOylation by silencing of SUMO-1 expression or mutations of the main α-syn SUMO-1 acceptor sites exacerbates METH-induced α-syn aggregation in neurons. In addition, UBC9 overexpression fails to promote clearance of METH-induced α-syn aggregation containing mutations in the major SUMOylation acceptor sites of α-syn. Moreover, a viable strategy to treat not only METH-induced neurotoxicity but also neurodegenerative diseases linked to the aggregation of α-syn may be to enhance the α-syn SUMOylation. Further study should focus on confirming the explicit mechanism of SUMOylation-regulated METH-related effects and the relationship between SUMOylation and degradation of α-syn through the UPS and the ALP in response to METH exposure.

## Ethics Statement

All procedures performed in studies involving animal participants conformed to the ethical standards of Ethics Committee of Nanfang Hospital, Southern Medical University and to the 1964 Helsinki Declaration and its later amendments or comparable ethical standards. There were no studies with human participants by any of the authors in the article.

## Author Contributions

LZ, HQ and LC were responsible for designing the experiment, completing the experiment and writing the article. PQ and BZ were responsible for directing and designing the experiment. LS and YL helped LZ and HQ with *in vitro* experiment. JH was major in virus production and establishment of stable expression in cells. JD and YM helped LZ and HQ with *in vivo* experiment. WX was responsible for controling the quality of experiment. LZ, HQ and LC contributed equally to this study.

## Conflict of Interest Statement

The authors declare that the research was conducted in the absence of any commercial or financial relationships that could be construed as a potential conflict of interest.

## Abbreviations

α-syn: alpha-synuclein
ALP: the autophagy-lysosome pathway
LAMP2A: Lysosomal Associated Membrane Protein 2A
METH: methamphetamine
PD: Parkinson’s disease
SENPs: SUMO-specific proteases
SUMO-1: the small ubiquitinrelated modifier 1
Ub: Ubiquitin
UPS: the ubiquitin-proteasome system.
