# Supplementary material for: SUMOylation of Alpha-Synuclein Influences on Alpha-Synuclein Aggregation Induced by Methamphetamine
Source: Front Cell Neurosci. 2018 Aug 24;12:262. doi: 10.3389/fncel.2018.00262 (PMC6117395; doi:10.3389/fncel.2018.00262)
Supplement: Supplementary file 1 [file Data_Sheet_1.doc]

**Supplementary Material**

**SUMOylation of alpha-synuclein influences on alpha-synuclein aggregation and neurotoxicity induced by methamphetamine**

Lin-nan Zhu^a,1^, Hong-hua Qiao^a,1^, Ling Chen^a,1^, Le-ping Sun^a^, Jia-liang Hui^b^, Yong-ling Lian^a^, Wei-bing Xie^a^, Jiu-yang Ding^a,c^ Yun-le Meng^a^, Bo-feng Zhu^a,*^, Ping-ming Qiu ^a,*^

^a^ School of Forensic Medicine, Southern Medical University, Guangzhou, China

^b^ First Clinical Medical College, Southern Medical University, Guangzhou, China

^c^ Department of anatomy, Zunyi medical college, Zunyi, China

^*^ Corresponding authors.

E-mail addresses: [qiupm@163.com](mailto:qiupm@163.com) (Ping-ming Qiu), [zhubofeng7372@126.com](mailto:zhubofeng7372@126.com) (Bo-feng Zhu)

1 These authors contributed equally to this study.

**Materials and** **methods**

*RT-QPCR*

Total RNA was extracted using RNAisoPlus Kit from SH-SY5Y cells with METH treatment for 24 h. Thereafter, cDNA was reversely transcribed from 1 mg of total RNA following the PrimeScriptTM RT reagent Kit and SYBRs Premix ExTaqTM Kit instructions. Primers were designed by Takara Bio Co., Ltd. (Dalian, China). All the sequences of proteins primers listed as Supplementary Table 1.

*Immunofluorescence Labeling*

For immunofluorescence staining of frozen tissue sections, all sample incubation solutions were prepared using phosphate-buffered saline (PBS) supplemented with 0.1% Triton X-100. VECTASHIELD Antifade Mounting Medium with DAPI (H-1200, VECTOR, Burlingame, USA) was used for nuclear labeling. Microphotographs were taken using a fluorescence microscopy (A1+/A1R+, Nikon, Tokyo, Japan). All digital images were processed using the same settings to improve the contrast.

**Results**

**Table 1**

Primer sequences for RT-PCR analysis.

| **Primer** | **Forward** **(5’-3’)** | **Reverse (5’-3’)** |
| --- | --- | --- |
| α-syn | GTGATATATATTGGGCGCTGGTGA | AGATGACACATGCAGCTTAGCACTC |
| SUMO-1 | ACCGTCATCATGTCTGACCA | TGGAACACCCTGTCTTTGAC |
| SENP-1 | TTGGCCAGAGTGCAAATGG | TCGGCTGTTTCTTGATTTTTGTAA |
| SAE1 | TTCTGAGTTGTTGCTCCACATACG | TGAGACAGGGCCTTCACAAT |
| UBC9 | CAGGAAAGAAAGGGACTC | TTCGGGTGAAATAATGG |
| PIAS1 | GGTATACGGGAAAAACCGG | TCAGAGGTTACGAGCAAAGG |
| GAPDH | GAAGGTGAAGGTCGGAGTC | GAAGATGGTGATGGGATTTC |


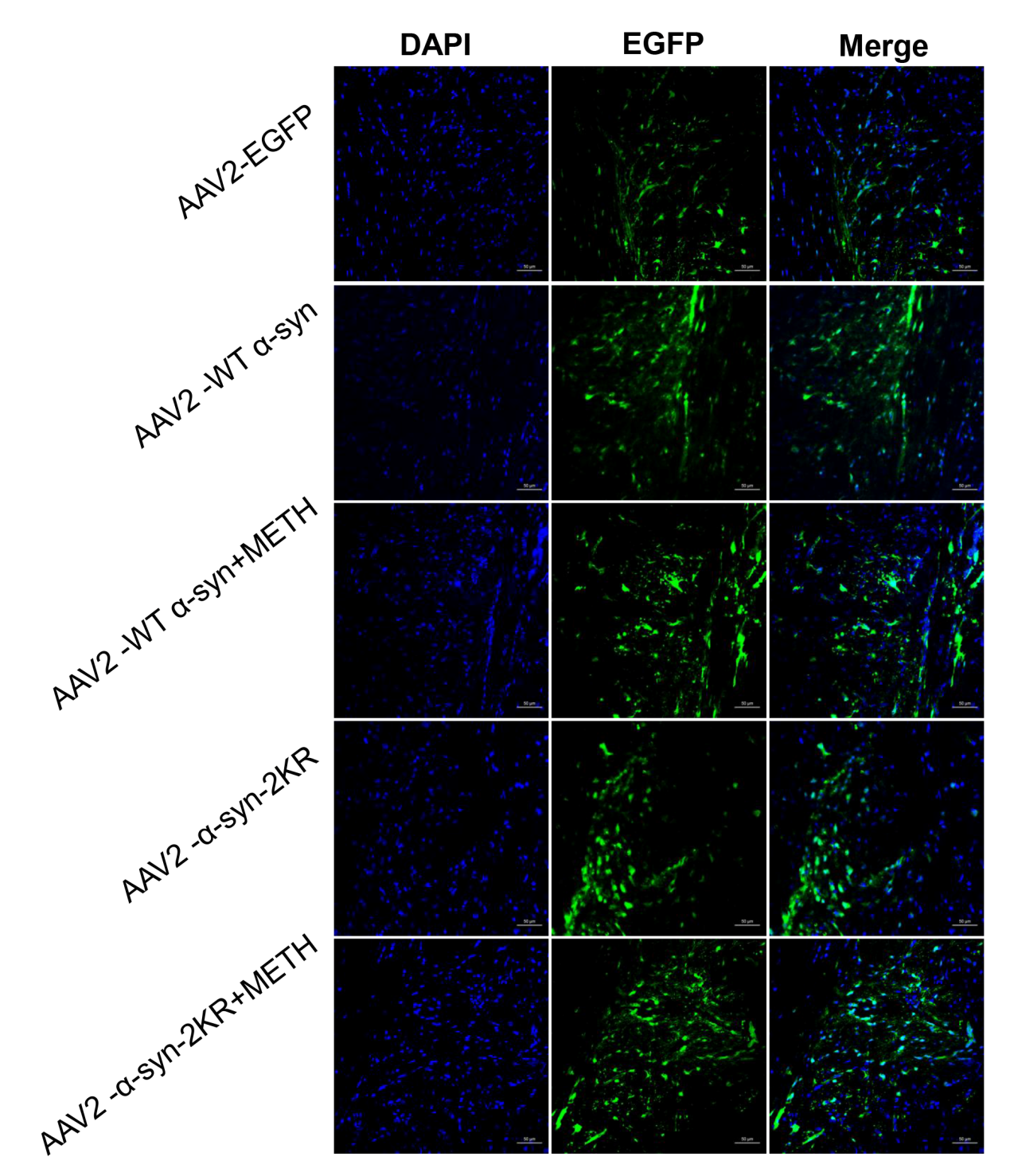


**Fig.1** Healthy adult male C57 BL/6 mice were injected with AAV2-EGFP, AAV2-WT α-syn and AAV2-α-syn-2KR using a stereotaxic injection system. Two days later, mice were exposed i.p. to saline or METH (8 injections, 15mg/kg/injection, at 12h intervals), and sacrificed 24h after the last injection. The brain samples were rapidly removed and the striatum were dissected on ice. Sections of striatum were used for fluorescence observation (magnification: 200x). Note that adenovirus vector was effectively delivered and expressed in the midbrain.
